# Recent Developments in Pharmacological Effect, Mechanism and Application Prospect of Diazeniumdiolates

**DOI:** 10.3389/fphar.2020.00923

**Published:** 2020-06-23

**Authors:** Bin Li, Yue Ming, Yao Liu, Haiyan Xing, Ruoqiu Fu, Ziwei Li, Rui Ni, Li Li, Dongyu Duan, Jing Xu, Chen Li, Mingfeng Xiang, Hongyu Song, Jianhong Chen

**Affiliations:** Department of Pharmacy, Daping Hospital, Army Medical University, Chongqing, China

**Keywords:** diazeniumdiolates, structure, characteristics, pharmacological effect, application prospect

## Abstract

Nitric oxide (NO) is a simple structured and unstable free radical molecule, which participates in the regulation of many pathophysiological processes. It functions both as a second messenger and as an endogenous neurotransmitter. Diazeniumdiolates (NONOates) are a series of compounds containing the functional parent nuclear structure of [N(O)NO]^—^, which are the most widely studied NO donors. NONOates are unstable and easy to release NO in physiological conditions. The biomedical applications and drug development of NO donor have attracted the scientists' attention in recent years. In this review, recent advances in NONOates research are highlighted in terms of chemical structures, molecular characteristics, pharmacological effects, and biomedical application prospects.

## Introduction

Nitric oxide (NO) is a simple and unstable free radical, which is considered as a versatile endogenous molecule and a crucial signaling factor in many biochemistry and physiological pathways. NO participates in such pathophysiological processes as vasodilation, signaling, and endocrine regulation (Heckler and Boon, 2019). On the one hand, depletion of NO or attenuation of its effector system may result in hypertension, angina and impotence. On the other hand, the overproduction of NO may cause many damages, such as circulatory shock, sepsis, neurodegenerative disorders and inflammatory responses (Stroppel et al., 2018; Spiller et al., 2019). NO can exist in three different redox states: as the uncharged form (NO•), as the nitroxyl anion (NO^−^) in the reduced state, and as the nitrosonium cation (NO^+^) in the oxidized state. They are mutually dependent and exchangeable. NO has unique pharmacokinetics as a potential therapeutic drug. It is small, gaseous and uncharged, and thus it can freely diffuse into tissues. However, the effects of NO are restricted locally due to its short half-life (approximately 1s) (Chiesa et al., 2018).

Because of the multiple roles of NO in pathophysiology, intense interest in the development of exogenous NO donor has been aroused. By modifying some functional groups in the molecule, different NO-donor compounds have been designed. Up to date, NO-donor compounds include diazeniumdiolates (NONOates), S-nitrosothiol, metal-nitrosyl, nitrobenzene, furoxans and oxadiazoles (Zhou et al., 2016). NONOate is one of the most extensively studied NO-donor compounds. Their biomedical applications and developments have attracted wide attention in relevant field (Sharma et al., 2018). This review summarizes some advances in NONOates about pharmacokinetic characteristics, pharmacological effects, mechanisms and application prospects in recent years.

## Diazeniumdiolates

Vascular endothelium is the main source of NO. The endothelial NO synthase (eNOS) regulates smooth muscle tone and platelet aggregation. The neuronal NO synthase (nNOS) generates NO in neuronal communication of cardiomyocytes regulating contractility. Both eNOS and nNOS are constitutively expressed generating basal NO concentration. The inducible NO synthase (iNOS) generates NO in macrophages, hepatocytes, and synoviocytes to regulate inflammation and immune functions. NONOate is considered as the effective substance for mimicking transient biosynthesis of NO, because its decomposition rate is mainly dependent on the amine component, pH and temperature. These different rates of NO generation make NONOates useful to mimic the low, transient NO synthesis by eNOS/nNOS.

NONOate is an optional, nucleophilic NO donor that offers many advantages as tools for probing the roles of NO in biological redox processes, especially in those physiological environment of requiring known, controllable rates of NO release. It is an exogenous NO donor agent with excellent performance developed in recent decades. NONOates are a series of compounds containing a X-[N(O)NO]^−^ functional parent nuclear structural unit. X is a secondary amine. They are synthesized by the reaction of secondary amine with NO gas (Keefer, 2005; Sharma et al., 2018) and some of them have been commercially available. NONOates are convenient and uniquely advantageous NO dosage forms. They are stable in solids state. However, NONOate compounds can rapidly self decompose when dissolved in aqueous solution, activate the soluble guanylate cyclase (sGC) system in organism to spontaneously generate up to two molecules of NO per [N(O)NO]^−^ unit. When X = O^−^, first-order dissociation produces NO^−^ rather than NO, but the ion becomes an NO source on 1-electron oxidation. NO derived from NONOate can also be used to generate reactive nitrogen/oxygen species with higher nitrogen oxidation states in the presence of selected oxidizing agents.

Interestingly, after the release of NO, there are no other metabolites produced except for the original amines. Since NONOate can produce different NO release rates and can be easily prepared into different physical states, there are local fast active drugs that can release NO completely within seconds, and long-term controlled-release drugs that need to be released for several hours. Therefore, NONOate is a kind of very important NO local release compounds. Researchers have developed NONOate macromolecule by developing amine-containing polymeric matrices to control the release of small molecule NONOates effectively (Zhang et al., 2019). These compounds have been widely explored in biomedical fields to show outstanding clinical application prospects. From the pharmaceutical point of view, it is very important that the design and modification of ionic NONOates can easily derive into other forms to deliver NO to the desired sites.

There are at least four characteristic advantages of NONOates, which make them the excellent agents in NO related research (Fitzhugh and Keefer, 2000). Firstly, NONOate have known rates of NO generation. NONOate are relatively stable in basic pH. They spontaneously decompose to generate 1–2 moles of NO per mole of donors with first order release kinetics in the presence of protons under physiological conditions. Secondly, the half-lives of NONOates exhibit a wide range with different chemical structures. The half-lives span ranges from 1.8 s (PROLI NONOate, at 37°C, pH 7.4) to 56 h (DETA NONOate, at 22–25°C, pH 7.4). Thirdly, NONOate spontaneously dissociate and release NO in solution. Most of them spontaneously split to release NO in a pH and temperture dependent manner, even without redox activation. Finally, three redox forms of NO can be present and exchangeable. Free radical (NO•) can be transformed into nitrosonium cation (NO^+^) or nitroxyl anion (HNO) by obtaining or losing an electron. Therefore, it is possible to use them as precursors of other NO donor compounds by activation and reaction. These attributes, such as namely structural diversity, dependable rates of NO release and a rich derivatization chemistry, make them designed to facilitate targeting of NO to specific need sites to solute the important clinical problems.

## Representative Drugs of Nonoate and Characteristics

NO inhibits platelet function and vasodilation. Hence, a majority of synthesized NO donor drugs are mainly used for treating cardiovascular diseases. The research intense in NO related drugs mainly focus on prodrugs, NO scavengers and inhibitors of endogenous NO synthesis. The goals of designing NO donor are to maximize the production of NO and to maintain the stability of drugs. Compared with other NO donors, NONOates don't require redox activation. They can release NO at repeatable first-order rates in the aqueous solution. On the other hand, NONOates have the broad array of reproducible NO generation rates. These fundamental chemistry features set the NONOate ions apart as advantageous tools for investigating NO. The predictability of NONOate on the NO release rate and its compatibility with a variety of compounds make it gain more significant advantages than most of other NO donor agents. Therefore, NONOate derivatives are one of the most widely investigated NO donor compounds, which are mainly developed as NO donor and HNO donor prodrugs. Such NONOates as DEA NONOate, SP NONOate, and DETA NONOate are commercially available. The structure, half-life and characteristics of representative of NONOates are summarized and listed in **Table 1**. In the future, more researches about NONOate might be focus on the field about its absorption through the skin and connection with polymer and plastic materials.

**Table 1:** The structure, half-life and characteristics of representative of NONOates.

Name	Molecular Formula	Molecular Weight	Half-life(t_1/2_)and Characteristics	Structure
PROLI NONOate (1-(hydroxy-NNO-azoxy)-L-proline, disodium salt) (Bahnson et al., 2016)	C_6_H_11_N_3_Na_2_O_5_	251.15	Half-life ≈1.8 s (at 37°C, pH 7.4) in aqueous solution, Dissociation is pH-dependent and first-order rates, which liberate 2 moles of NO/mole of PROLI NONOate.	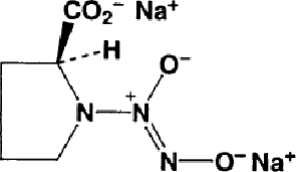
MAHMA NONOate (Methylamine hexamethylene methylamine NONOate) (Homer and Wanstall, 2003)	C_8_H_20_N_4_O_2_	204.27	Half-life ≈1 minute (at 37°C, pH 7.4) and 3 min (at 22-25°C, pH 7.4) in aqueous solution, respectively. 2 moles of NO is liberated from 1 mole of MAHMA NONOate.	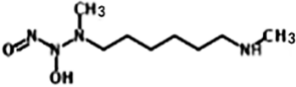
DEA NONOate (Diethylamine NONOate) (Turgut et al., 2013)	C_4_H_10_N_3_NaO_2_	155.13	Half-life ≈2min (at 37°C, pH 7.4) and 16 min (at 22-25°C, pH 7.4). 1.5 moles of NO is liberated from 1 mole of DEA NONOate in aqueous solution.	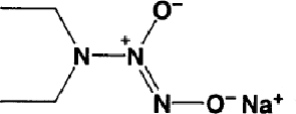
PAPA NONOate (Propylamine Propylamine NONOate) (Bobko and Khramtsov, 2014)	C_6_H_16_N_4_O_2_	176.22	Half-life ≈15 min (at 37°C, pH 7.4) and 77 min (at 22-25°C, pH 7.4) in aqueous solution. 1 mole of PAPA NONOate is dissociates to liberate 2 moles of NO.	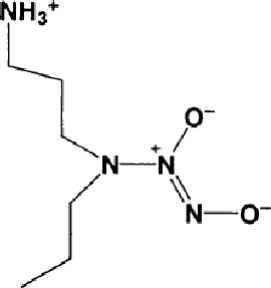
DPTA NONOate (Dipropylenetriamine NONOate) (Dabrowska et al., 2011)	C_6_H_17_N_5_O_2_	191.23	Half-life is about 3 h (at 37°C, pH 7.4) and 5 h (22-25°C, pH 7.4), pH 7.4, Dissociation is a pH-dependent, first-order process. 1 mole of DPTA NONOate is dissociates to liberate 2 moles of NO.	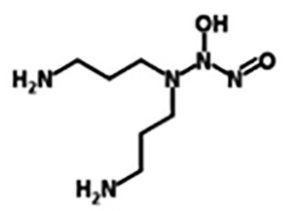
Spermine NONOate/SP NONOate ((Z)-1-[N-[3-aminopropyl]-N- [4-(3-aminopropylammonio) butyl]-amino]diazen-1-ium-1,2-diolate) (Ertug et al., 2014)	C10H26N6O2	262.35	Half-life of is about 39 min (at 37°C, pH 7.4) and 230 min (22-25°C, pH 7.4), respectively, Dissociation is first-order rate and pH-dependent process. 1 mole of SP NONOate is dissociated to liberate 2 moles of NO.	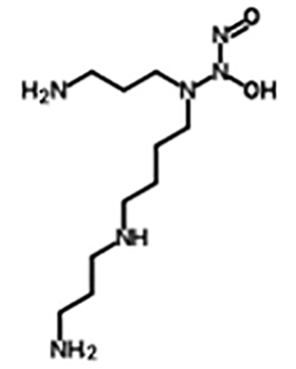
DETA NONOate (Diethylenetriamine NONOate) (Waheed et al., 2019)	C4H13N5O2	163.18	Half-life of is about 20 h (37°C, pH 7.4) and 56 h (at 22-25°C, pH 7.4), respectively, 2 moles of NO is liberated from 1 mole of DETA NONOate.	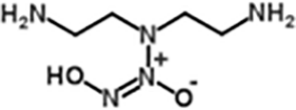
β-Gal-NONOate (1-O-(1-pyrrolidinyl-ONN-azoxy)-beta-D-glucopyranose) (Chen et al., 2006a)	C10H19N3O7	293.27	Half-life is about 6 min (at pH 5.6), β-Gal-NONOate is stabile at neutral for several h in aqueous solution and actived by β-galactosidase to release NO.	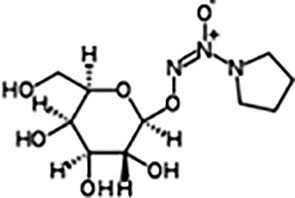
Sulpho NONOate (Disodium (E)-1-sulfonatodiazen -1-ium-1,2-diolate) (Rivera-Tirado et al., 2011)	H10N4O5S	178.17	Sulpho NONOate does not produce NO at physiological pH. Therefore, it usually may be used in the experiments about other NO-donor regents as a negative control.	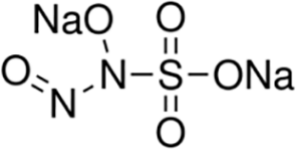
IPA NONOate (Isopropylamine NONOate) (Miranda et al., 2005)	Na[(CH3)2CHNH(N(O)NO]	141.10	Half-life is about 2 min in solution. At physiologic pH, HNO and NO is the main products released from the breakdown of IPA NONOate. The releasing level depends on the reaction conditions such as buffer pH and initial concentration of IPA NONOate.	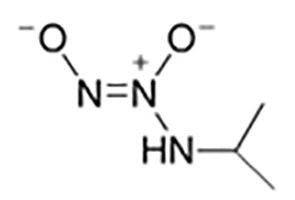
Ni(PipNONO)Cl (nickel-piperazine/NO donor compound) (Ziche et al., 2008)	C6H14N5O2NiCl	282.35	Ni(PipNONO)Cl is a kind of metal-nonoates, half-life is about 4.8min, the rate constant of NO release is (2.41±0.05)′10^−3^s^−1^	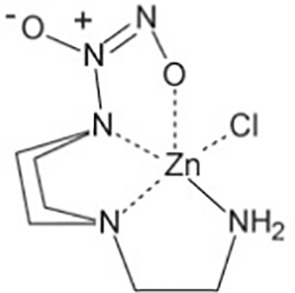
Zn(PipNONO)Cl (zinc-piperazine/NO donor compound) (Monti et al., 2018)	C6H14N5O2ZnCl	289.05	Zn(PipNONO)Cl is another metal-nonoate. Half-life is about 385s. Zn(PipNONO)Cl show very similar NO-releasing properties with Ni(PipNONO)Cl, while zinc is less toxic than nickel. The absorption profile of Zn(PipNONO)Cl followed a simple exponential curve, the rate constant of NO release is (1.79±0.01)'10^-3^s^-1^	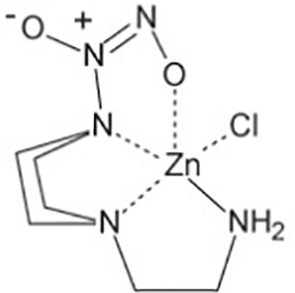

## Pharmacological Activity and Development Prospect of Nonoate

### Effects on Cardiovascular System

In terms of cardiovascular system function, NO can relax vascular smooth muscle and inhibit platelet function. Therefore, NO related drugs, including NO donor prodrugs, NO scavengers and endogenous NO synthesis inhibitors, can be applied in the treatment of cardiovascular system diseases. Herein, we firstly review the pharmacological effects, mechanisms and application prospects of NONOates in cardiovascular diseases.

#### Vasorelaxation

NO is one of the most important vasodilator active substances *in vivo*. The vasodilator effect of NO donors provides a great potential for the treatment of such cardiovascular diseases as hypertension, angina pectoris and congestive heart failure. SP NONOates caused the expansion of femoral artery in rats by increasing the level of extracellular NO through the release after cleavage although its vasodilation is only partially mediated by soluble guanylate cyclase (sGC). It is suggested that the activity of NO and NO donors is not completely mediated by sGC (Miller et al., 2004). As an oral drug for the treatment of erectile dysfunction (ED), Sildenafil (Viagra) enhanced the vasodilatory effect of NO by specific inhibition of phosphodiesterase 5 (PDE5) to decrease the decomposition of cGMP. It is an oral drug for the treatment of erectile dysfunction (ED). SP NONOate is capable of potent relaxant action in the smooth muscle of penile cavernous body, which can be enhanced through oral sildenafil treatment. Therefore, SP NONOate is also considered as a potential drug for ED treatment (Ertug et al., 2014). Pinkney et al. demonstrated that IPA NONOate inhibited the hyperpolarization and contraction of arterial smooth muscles induced by phenylephrine, an α 1-adrenoceptor agonist, in a concentration dependent manner (Pinkney et al., 2017). Moreover, DETA NONOate might be beneficial for the outcome of coronary artery bypass grafting by producing continuous and long-lasting NO. This finding suggests that DETA NONOate has a significant relaxative effect (Rahimi et al., 2011).

#### Vascular Protective Activity

The production of reactive oxygen species or superoxide anion is one of the pathogenic features of cardiovascular disease. NADPH oxidase can boost oxidative stress of vasculature in atherosclerosis. Excessive superoxide in arteries directly inactivates NO in endothelium and damages its vasoprotective effects. Selemidis found that the suppressive effect of some new NONOates on superoxide production was dependent on vascular NADPH oxidase making them more suitable for atherosclerosis therapy (Selemidis, 2008). Metal-NONOate Zn(PipNONO)Cl protected vascular endothelium by producing endogenous H2S and NO (Monti et al., 2018). DETA NONOate could completely block the release of superoxide dismutase and the expression and activity of PDE5 protein in vascular smooth muscle cells (VSMCs). PDE5 could attenuate the vascular protection effect of NO through hydrolyzing cGMP (Muzaffar et al., 2008).

#### Reduction of Neointimal Hyperplasia and Atherosclerosis

As a one-electron reduction form of NO, HNO exhibited biological and pharmacological effects distinct from NO. IPA NONOate is a HNO donor at physiologic pH. IPA NONOate moderately inhibited intimal hyperplasia by reducing the proliferation of VSMCs and macrophage infiltration. In rat carotid artery injury model, IPA NONOate also inhibited endothelial cell proliferation (Tsihlis et al., 2010). In hypercholesterolemic mice, IPA NONOate presented both vasorelaxant effect and antiplatelet aggregation whereas the inhibitory effect of glyceryl trinitrate on platelet aggregation was abolished (Bullen et al., 2011).

In the early development of atheroma-like lesions, the thickness of the neointima in arterial collared artery was significantly reduced (by 74%) in rabbits treated with SP NONOate in comparison with control animals after 14 days with SP NONOate treatment. Meanwhile, the treatment did not influence the blood pressure of mice. These results suggest that local administration of SP NONOate could effectivelly slow the development of neointima in this model (Yin and Dusting, 1997). Low concentrations of SP NONOate had minor inhibitory effect on low-density lipoprotein (LDL) oxidation while high concentration (> 4mM) showed stronger inhibition. NO directly interacted with lipid peroxides (LPO) to form lipid nitroso adducts. This product could eliminate free LPO radicals and eliminate both free LPO radicals and the regeneration of LPO, thus preventing further aggravation and transition (Goss et al., 1995).

Therefore, as HNO/NO donor, the application and safety of IPA NONOate on cardiovascular diseases such as congestive heart failure (CHF) have been extensively conducted.

#### Inhibition of Platelet Function and Vasodilating Effects

NO is a physiological inhibitor of platelet aggregation. MAHMA NONOate inhibited the aggregation of collagen and ADP in platelet rich plasma (Homer and Wanstall, 2003). Low concentration of MAHMA NONOate activated the platelet inhibitory systems and decrease platelet aggregation in a cGMP dependent manner rapidly and transiently. This effect appeared to be cGMP independent at a high concentration (Kobsar et al., 2014). MAHMA NONOate inhibited the rat platelet aggregation by activation of sarco-endoplasmic reticulum calcium ATPase. This inhibitory effect was largely ODQ (a soluble guanylate cyclase inhibitor) resistant (Homer and Wanstall, 2002). The effect of MAHMA NONOate on platelet inhibition was approximately 10-fold more potent than S-nitrosoglutathione (GSNO). It could also reduce systemic arterial pressure more than GSNO. Therefore, *in vivo*, MAHMA NONOate had platelet inhibition and vasodepressor effects (Homer and Wanstall, 2003).

#### Reduction in Pulmonary Hypertension

DPTA NONOate attenuated pulmonary hypertension induced by group B Streptococcus. The vasodilation effect of DPTA NONOate in pulmonary vessels was more visible than that in systemic vessels (Dabrowska et al., 2005; Dabrowska et al., 2011). DEA NONOate relaxed the isolated rat small mesenteric artery by cGMP dependent/independent mechanisms (Turgut et al., 2013). SP NONOate relaxed rat pulmonary artery by producing cGMP, which could regulate the activation of Na^+^/K^+^-ATPase, Ca^2+^-ATPase and Ca^2+^-activated K^+^ channels (Homer and Wanstall, 2000). Jahidur Rashid et al. reported that a liposome was loaded both fasudil (a rho kinase inhibitor) and DETA NONOate to treat pulmonary hypertension (PAH). The results showed that the formulation of the liposome (loaded fasudil and DETA NONOate) could increase the level of cGMP in pulmonary artery muscle cells (SMCs) of PAH patients, which suggest that this combination therapy might be an effective treatment for PAH (Rashid et al., 2018).

#### Anti Cardiac Hypertrophy Effect

IPA NONOate inhibited the increase in cardiomyocyte size induced by endothelin-1 (ET1). IPA NONOate also alleviated myocardial hypertrophy induced by chronic pressure overload. The protective mechanism of IPA NONOate against cardiomyocyte hypertrophy was *via* HNO activation of cGMP signaling (Irvine et al., 2013).

#### Inhibition of Matrix Metalloproteinase-9-Dependent Remodeling on Vessel Wall

DETA NONOate adminstration increased NO concentration in rat aortic SMCs and explants. The expression of MMP-9 induced by IL-1β could be decreased by DETA-NONOate. In addition, there were no significant effects on the expression and activity of MMP-2 and tissue inhibitor of matrix metalloproteinases-1 in response to DETA NONOate treatment. These results indicate that DETA-NONOate may be helpful to inhibit the MMP-9-dependent vessel wall remodeling during abdominal aortic aneurysm formation (Sinha et al., 2006).

#### Angiogenesis

NO is an important angiogenic regulator. In angiogenic experiments and clinical research, compounds of NONOate subfamily have been used as tool agents. Majumder et al. reported that NO donors with different release patterns have different regulatory effects on angiogenesis, suggesting that SP NONOate had an unique NO release pattern which is most suitable for angiogenesis (Majumder et al., 2014). NO is necessary for arteriogenesis but iNOS also plays an important part. DETA NONOate potently stimulated the growth of collateral artery, activated perivascular monocytes, and increased the expression of proliferation markers in vessel cells. Shear stress-induced NO may activate the innate immune system and iNOS (Troidl et al., 2010). Metal-diazeniumdiolate complexes {[Cu(PipNONO)Cl] and [Ni(SalPipNONO)]} effectively induced vasorelaxation and endothelial cell proliferation (Ziche et al., 2008).

Although NO has a wide range of effects on cardiovascular diseases, some studies found that continuous low concentration inhalation of NO might produce a significant effect, and increasing NO concentration did not improve the clinical efficacy. Moreover, the high concentration of NO may be leading to cytoxicity and DNA damage. Therefore, how to balance the clinical application of NO donor compounds and the discovery of novel compounds will be the key to NO donor drug research.

### Effect on Nervous System

#### Neuroprotection

In the central nervous system, NO displays a series of functions, such as promoting the release of neurotransmitters, participating in the processes of synaptic plasticity and pain modulation, regulating the sleep wake cycle, etc. In the peripheral nervous system, NO is considered to be a neurotransmitter or mediator of non-cholinergic and non-adrenergic nerves, participating in the process of pain afferent and sensory transmission. Hua et al. (2008) demonstrated that DETA NONOate reduced the hippocampal neurogenesis impairment induced by chronic mild stress (CMS) and reversed behavioral damage, suggesting that NONOate could be applied in the treatment of chronic stress depression by stimulating the neurogenesis of hippocampus tissue

#### Neurotoxicity

Physiological amounts of NO are neuroprotective, whereas higher concentrations are clearly neurotoxic (Hua et al., 2008). NO will become noxious if it is excessively produced especially under redox reaction condition and toxic compounds reactive nitrogen species (RNS) will be formed to cause cell damage. In the development and progression of neurodegenerative disorders, NO and RNS demonstrate a pathogenetic effect (Balez and Ooi, 2016). NO has been considered as a part of the neurotoxic insult caused by neuroinflammation in the Alzheimer's brain. However, this perception is changing due to recent researches in the early developments of Alzheimer's disease, especially prior to the appearance of cognitive symptoms. NO could compensatorily protect synapses by increasing neuronal excitability. This neuroprotective mechanism of NO was *via* modulation of voltage-gated potassium channel activity (Hua et al., 2008).

#### Anti-Oxidative Damage

NO induced the function and expression of heme oxygenase-1 (HO-1), a key enzyme in cell stress response. NO is considered as an antioxidant and had a direct neuroprotective effect on oxidative damage. In addition, NO is involved in cell protection by activating sGC to increase cGMP concentration. DETA NONOate (1-10 mM) inhibited the neurotoxicity caused by H_2_O_2_ (20-100 mM) in 1-week-old rat cortical neurons (Fernandez-Tome et al., 1999).

#### Reverse Activation of Inward Current in Neurones

Thompson et al. (2009) demonstrated that DETA NONOate induced an inward current in cerebellar granule cells by directly activating cation selective channels through a whole cell patch clamp technique. However, GSNO, another NO donor, led to outward current generation in these cells. The interpretation and judgment of the results need further investigation when high concentrations of these NO donor compounds are used (Porshneva et al., 2018).

#### Traumatic Brain Injury and Neurodegenerative Disorders

In the rat model of traumatic brain injury (TBI) model rats, DETA NONOate significantly increased the survival, proliferation, differentiation and migration of neural progenitor cells in the injured cortex, hippocampus and other brain tissues. In addition, it improved the neurological function significantly. These results indicate that DETA NONOate has significant benefits for the treatment of TBI (Lu et al., 2003).

The role and application of NO donor compounds on the prevention of neurodegenerative diseases are still unclear. Because the drugs acting on the NO-cGMP pathway are able to counteract NO-induced damage, some scientists have proposed to use NO donor drugs to prevent or treat Alzheimer's disease. However, the pharmacokinetic characteristics of these substances may limit their uses in neurodegenerative diseases so more research work will be required in the future.

### Anti Tumor Effects

#### Inhibition of Tumor Growth, Invasiveness, and Angiogenesis

Although NO donors have been used for antitumor therapy, the effects of NO on growth and progression of tumors depends on its concentration. In 2006, Chen et al. (2006a) reported that β-Gal-NONOate only released NO when hydrolyzed by induced β-galactosidase in 9L/LacZ cells, which led to its more powerful cytotoxicity than that of NONOate. However, in 9L cells, β-Gal-NONOate showed less toxicity than NONOate. Therefore, β-Gal NONOate is a promising prodrug for targeting intracellular NO, which is expected to become a potential therapeutic drug for cancer ETA/NO dose- and time-dependently induced the cell viability of human endometrial tumors. The mechanism is activating caspase-3 and arresting cell cycle at the G0/G1 phase, associated with attenuating the expression of cyclin-D1 and D3 (Waheed et al., 2019).

A metal-nonoate Ni(SalPipNONO) inhibited the cell proliferation and invasion of A549 cells (human lung carcinoma cells) to promote apoptosis. The antitumor activity of Ni(SalPipNONO) was more effective than that of DETA NONOate. The antitumor mechanism of Ni(SalPipNONO) was partly due to NO-cGMP dependent pathway, and partly to the salicylaldehyde moiety and ROS activated ERK1/2 signaling converging on p53 dependent caspase-3 cleavage. Furthermore, Ni(SalPipNONO) inhibited the angiogenesis of A549 cells, which was associated with reducing angiogenic factor expression and endothelial cell functions (Ciccone et al., 2018).

In general, these findings confirm that NO donor compounds produce its antitumor effect through a variety of mechanisms. NONOates can be further developed as a single agent or combination therapy.

#### Synergistic Effect With Chemotherapy

Treatment with NO donor enhanced the sensitivity of various tumors to chemotherapy, radiotherapy and immunotherapy. The potential mechanism of immune resistance in tumor is related to the anti proliferation and anti apoptosis pathways, as well as the increased expression of transcription factor Yin-Yang-1 (YY1) and programmed death ligand-1 (PD-L1). Overcoming these resistance mechanisms are needed to improve the sensitivity of anti-tumor therapy. NO donor decreased the expression of YY1 and PD-L1 by inhibiting the upstream NF-κB pathway. NO donor inhibited the S-nitrosylation of YY1 and then led to the inhibition of YY1 expression and the binding activity of YY1-DNA (Hays and Bonavida, 2019). β-Gal-NONOate enhanced the sensitivity of C6/lacZ, 9L/lacZ or HeLa/lacZ tumor cells to cisplatin, and reduced the dosage and side effects of cisplatin. Therefore, β-Gal-NONOate can be used as a chemosensitizer in tumor treatment with bright application (Deng et al., 2013).

As shown in above research, NONOates can exert the anti-tumor efficiency through multiple mechanisms and synergistic anti-tumor as a chemosensitizer, reduce or reverse drug resistance. Therefore, the development and research of NONOates has a good prospect as the novel antitumor agents.

### Anti-Bacterial Effect

#### Bactericidal Activity

In the *Escherichia coli* transformed with lacZ gene, β-Gal-NONOate showed a stronger bactericidal effect than conventional NONOate. While, in the *E. coli* without lacZ gene, both β-Gal-NONOate and NONOate presented a low bactericidal activity. In the acute model of Pseudomonas aeruginosa (*Pseudomonas aeruginosa*) pneumonia, intermittent nebulization of DETA NONOate prolonged the continuous intrapulmonary delivery of NO and decreased the bacterial number in the lungs of model mice. However, there is no obvious influence on the infiltration of pulmonary leukocytes. Although the number of bacterial colonies was reduced after exposure to DETA NONOate *in vitro*, the antibacterial effect of NONOate was closely related with its nucleophilic backbone instead of NO. This finding indicates that the therapeutic effect of exogenous NO on *P. aeruginosa* pneumonia is limited (Dukelow et al., 2002).

#### Synergistic Antibacterial Effects

Bacterial biofilm is one of the important causes of bacterial resistance, chronic and recurrent infection. Rashin et al. (2018) reported that a novel NO-loaded polymer was synthesised. The NO-loaded polymer consisted of biocompatible oligoethylene glycol, hydrophobic ethylhexyl, cationic primary amine and NO donor. It exerted synergistic antibacterial effects through the eradication of biofilm mediated by NO and the disruption of *P. aeruginosa* cell membrane assisted by cationic polymer to induce bacterial death.

### Other Effects

#### Acceleration of Wound Healing

NO plays a pivotal role in the cutaneous healing process. The topical supplement of NO is beneficial for wound healing. Zhang et al. reported a novel polyethylenimine (PEI) based NO donor polymer (PEI-PO-NONOate polymer), which presented high loading efficiency and controllable NO releasing curve and topical supplement of NO to promote wound healing. PEI-PO-NONOate polymer increased the formation of granulation tissue, collagen deposition and angiogenesis. Therefore, it has potential application prospects in accelerating the healing of cutaneous wound (Zhang et al., 2019).

#### Relieving of Ischemia/Reperfusion Injury in Transplanted Organs

Ischemia/reperfusion injury of transplanted organs may be associated with the loss of endothelial release of NO. Compared with control group, DEA NONOate significantly enhanced all hemodynamic parameters (12 h later) and aortic blood flow (6 h later). DEA NONOate treatment significantly improved coronary blood flow and cardiac function of rat isolated heart after 12 h of cryopreservation. These results indicate that the compounds have new applications in transplantation medicine (Kai et al., 2016).

#### Nonalcoholic Steatohepatitis

A variety of external or internal factors, such as inflammatory stimulation, oxidative stress and cytokines, could cause the activation of M1 macrophages in nonalcoholic steatohepatitis (NASH). Cytochrome P450 2E1 (CYP2E1) was closely associated with the polarization of M1 macrophages. Administration of NO donor DETA NONOate mechanistically inhibited CYP2E1 catalyzed the oxidative stress during the study in NASH-abrogated M1 polarization and NASH progression. This finding suggests that DETA NONOate is a promising strategy to the treatment of NASH (Seth et al., 2015).

#### Inhibition of Activation of Mast Cells

Exogenous NO had inhibitory action on human mast cells. IgE induced the release of histamin by mast cell. DEA NONOate dose-dependently inhibited histamine, eicosanoids and cytokines release induced by IgE. Regulation of NO by human mast cells was associated with the activation of the sGC-cGMP-PKG pathway and the inhibition of MAPK and NF kappaB phosphorylation (Yip et al., 2010).

#### Stimulation of Cl^—^ Secretion by Airway Cells

Airway cells absorbed Na^+^ and secreted Cl^−^ and bicarbonate ions *via* active transport mechanisms. The osmotic forces created by the coordinated transport of these ions are responsible for the movement of fluid across airways. NO stimulation of sGC was sufficient and necessary for increasing of short-circuit currents *via* stimulation of the apical cystic fibrosis transmembrane regulator (CFTR). Low concentration of NO donor DETA NONOate (1–1,000 μm) may help stimulate airway cells to secrete Cl^−^, but has no influence on alveolar edema (Chen et al., 2008).

#### Inhibition of Oxygen Consumption of Rat Leydig Cells *In Vitro*

NO plays an important role in the regulation of male reproductive system. NO and NOS regulated the secretion of androgen and sperm function. DPTA NONOate (10^−3^–10^−6^ M) inhibited oxygen consumption in isolated rat testis interstitial cells in a concentration dependent manner (Kojic et al., 2008).

#### Reducing Adhesions Formation in Rat Uterine Horn Model

Postsurgical adhesions occur following virtually all types of surgery, resulting in serious clinical complications. Endometrium adhesion is the most important cause of sterility. In rat uterine horn model, the adhesion formation at the site of peritoneal injury was significantly reduced by postoperative administration of N,O-carboxymethylchitosan and SP NONOate (Duran et al., 2003).

## Summary and Conclusions

The finding of NO about the multiple physiological and pathophysiological functions has promoted a great number of pharmaceutical researches to develop NO-related agents for therapeutic purposes. In particular, NO-donors prodrugs may have some important therapeutic effects on such diseases as cardiovascular diseases (arterial hypertension, atherosclerosis, platelet aggregation, cardiac hypertrophy and male sexual impotence etc), nervous system diseases (traumatic brain injury, neurodegenerative disorders etc), tumor, bacterial infection and inflammatory, immune or endocrine diseases. Intense pharmacological investigations in a series of NONOates are underway. Each NONOate has a specific pharmacokinetic and pharmacodynamic characteristic. The versatile roles of NONOate compounds, such as neuroprotective, cardioprotective, oncoprotective and immunoprotective agents, emphasize their important place in modern drug design and discovery. The effect, mechanism and application prospect of NONOates is summarized in **Table 2**. Although a growing number of novel NONOate compounds are synthesized and reported on their properties by different laboratories. Our view is that the clinical potential of NONOates should be maximized through a deeper and boarder understanding about their fundamental chemistry characteristics, structure, analytical and reactivity studies, mechanisms of NO release and their redox behavior, etc. The goal of expanding this knowledge base is worth of engagement of more laboratories and scientists. In the future, this field might be needed much more efforts and imagination to explore the basic chemistry charactristics of the NONOate compounds to design and gain the more improved tools for chemical biology research, and important advances in medical engineering practice.

**Table 2:** The effect, mechanism and application prospect of NONOates.

Compound	Pharmacological effect	Mechanism	Application prospect
DETA NONOate	Vasorelaxation	Continuous and long-lasting NO production (Rahimi et al., 2011)	Coronary artery bypass grafting
DETA NONOate	Vascular protection	Blocks SOD release and PDE5 expression (Muzaffar et al., 2008)	Atherosclerosis
DETA NONOate	Inhibition of MMP-9-dependent vessel wall remodeling	Decreases MMP-9 expression and activity induced by IL-1β (Sinha et al., 2006)	Abdominal aortic aneurysm formation
DETA NONOate	Angiogenesis	Activates the innate immune system and activate inos (Troidl et al., 2010)	Ischemic disease
DETA NONOate	Neuroprotection	Activates Akt and cAMP-responsive- CREB (Balez and Ooi, 2016)	Nervous system injury
DETA NONOate	Neurotoxicity	Produces toxic compounds RNS (Balez and Ooi, 2016)	Neurodegenerative disorders
DETA NONOate	Anti-oxidative damage	Activates soluble guanylate cyclase and increased cGMP concentrations (Fernandez-Tome et al., 1999)	Oxidative damage in cortical neurons
DETA NONOate	Reverse activation of an inward current in neurons	Activates cation-selective channels (Porshneva et al., 2018)	
DETA NONOate	Traumatic brain injury and neurodegenerative disorders	Enhances the proliferation, differentiation and migration of neural progenitor cells of injured cortex (Lu et al., 2003)	Traumatic brain injury Neurodegenerative disorders
DETA NONOate	Bactericidal activity	Decreases pulmonary bacterial load in mice with pneumonia (Dukelow et al., 2002)	Pneumonia, *P.aeruginosa* infection
DETA NONOate	Liver protection	Inhibition of CYP2E1-catalyzed oxidative stress (Seth et al., 2015)	Nonalcoholic steatohepatitis
DETA NONOate	Antidepressant effect	Reverses hippocampal neurogenesis impairment induced by CMS (Hua et al., 2008)	Depression
DETA/NONOate	Anti tumor effect	Attenuates cyclin-D1 and D3 expression (Waheed et al., 2019)	Endometrial tumors
DETA NONOate	Stimulation of Cl^−^ secretion	Stimulates sGC, resulting in increased cGMP levels and activation of PKGII, leading to phosphorylation and activation of CFTR (Chen et al., 2008)	Respiratory disease
SP NONOate	Slowing the development of neointima	Removes the lipid peroxyl radical but also abolish the regeneration of lipid hydroperoxide (Goss et al., 1995)	Atherosclerosis
SP NONOate	Relaxant action in cavernous tissue	Releasing extracellular no (Ertug et al., 2014)	Erectile dysfunction
SP NONOate	Reduction in pulmonary hypertension	Producing cGMP to relax pulmonary artery (Homer and Wanstall, 2000)	Pulmonary hypertension
SP NONOate	Anti tumor effect	Increases NO sensitive sites in heparan sulfate and polyamine uptake (Cheng et al., 2009)	Cancer
SP NONOate	Anti-inflammatory effect	Reducing adhesions formation in rat uterine horn model (Duran et al., 2003)	Sterility
IPA NONOate	Reduction of neointimal hyperplasia	Inhibits endothelial cell proliferation (Tsihlis et al., 2010) and platelet aggregation (Bullen et al., 2011)	Atherosclerosis
IPA NONOate	Anti cardiac hypertrophy effect	Inhibited the increasing of cardiomyocyte size induced by ET1; HNO activated cGMP signaling (Irvine et al., 2013)	Myocardial hypertrophy
DPTA NONOate	Reduction in pulmonary hypertension	Produces cGMP, activated Na^+^/K^+^-ATPase, Ca^2+^-ATPase and Ca^2+^-activated K^+^ channels (Homer and Wanstall, 2000)	Pulmonary hypertension induced by group B Streptococcus
DPTA/NO	Inhibition of oxygen consumption	Inhibition of oxygen consumption of rat Leydig cells *in vitro* (Kojic et al., 2008)	Tissue hypoxia; Sterility
DEA NONOate	Reduction in pulmonary hypertension	Relaxes isolated rat small mesenteric artery (Turgut et al., 2013)	Pulmonary hypertension
β-Gal-NONOate	Anti tumor effect	Inhibition of tumor growth, invasiveness and angiogenesis (Chen et al., 2006a)	Glioma
β-Gal-NONOate	Bactericidal activity	Decreases pulmonary bacterial load (Chen et al., 2006b)	*E. Coli* with transferred lacz gene
DEA NONOate	Ischemia/reperfusion injury	Improvement of coronary blood flow and heart function (Kai et al., 2016)	Organ transplant
DEA NONOate	Inhibition of activation of mast cell	The activation of the sGC-cGMP-PKG pathway and the inhibition of MAPK and NF kappaB phosphorylation (Yip et al., 2010)	Allergy
Gal-NONOate	Synergistic effect with chemotherapy	Inhibits of YY1 and PD-L1 expression (Hays and Bonavida, 2019)	Drug resistance of tumor
MAHMA NONOate	Platelet inhibitory and vasodepressor effects	Activates sarco-endoplasmic reticulum calcium ATPase (Homer and Wanstall, 2002)	Atherosclerosis
NONOate-based polymeric NO donors	Synergistic antibacterial effects	Eradicates biofilm and disrupted bacterial membrane (Rashin et al., 2018)	Infectious disease
Zn(PipNONO)Cl	Vascular endothelium protective effect	Produces endogenous H2S and NO and suppressed nadph oxidase dependent oxidative stress (Monti et al., 2018)	Atherosclerosis
Ni(SalPipNONO)	Anti tumor effect	Inhibits clonogenicity and cell invasion, promoted apoptosis(Ciccone et al., 2018)	Lung carcinoma
PEI-PO-NONOate	Accelerates wound healing	Enhances collagen deposition, granulation tissue formation, and angiogenesis (Zhang et al., 2019)	cutaneous wound healing

## Author Contributions

All authors participated in collecting and discussing of literature data. BL and YM drafted the manuscript. YL and HX summarized and drew the tables. RF revised the spelling and grammar. ZL, RN, LL, DD, JX, CL, MX, and HS participated in data extraction. JC critically reviewed the review and made suggestions for improvements. All authors read and approved the final manuscript.

## Funding

This study was supported by the financial support from National Science and Technology Major Project (Grant NO. 2018ZX09J18109-005). We also gratefully acknowledge the financial support from Logistics Application Fundamental research key project (Grant NO. BWS17J031-08) and the Science and Health Joint Medical Research Project of Chongqing (Grant No. 2018MSXM002). We thank Mr. Yuanyuan Li for technical assistance.

## Conflict of Interest

The authors declare that the research was conducted in the absence of any commercial or financial relationships that could be construed as a potential conflict of interest.
